# Molecular diet analysis of neotropical bats based on fecal DNA metabarcoding

**DOI:** 10.1002/ece3.7579

**Published:** 2021-05-02

**Authors:** Melissa R. Ingala, Nancy B. Simmons, Claudia Wultsch, Konstantinos Krampis, Kaiya L. Provost, Susan L. Perkins

**Affiliations:** ^1^ Division of Mammals Department of Vertebrate Zoology National Museum of Natural History Smithsonian Institution Washington DC USA; ^2^ Richard Gilder Graduate School The American Museum of Natural History New York NY USA; ^3^ Department of Mammalogy, Division of Vertebrate Zoology The American Museum of Natural History New York NY USA; ^4^ Division of Invertebrate Zoology The American Museum of Natural History New York NY USA; ^5^ Sackler Institute for Comparative Genomics The American Museum of Natural History New York NY USA; ^6^ Bioinformatics and Computational Genomics Laboratory Hunter College City University of New York New York NY USA; ^7^ Department of Biological Sciences Hunter College City University of New York New York NY USA; ^8^ Institute of Computational Biomedicine Weill Cornell Medical College New York NY USA; ^9^ Department of Ornithology The American Museum of Natural History New York NY USA; ^10^ Department of Evolution, Ecology and Organismal Biology The Ohio State University Columbus OH USA

**Keywords:** community ecology, diet analysis, DNA barcoding, mammals, tropical ecology

## Abstract

Bat communities in the Neotropics are some of the most speciose assemblages of mammals on Earth, with regions supporting more than 100 sympatric species with diverse feeding ecologies. Because bats are small, nocturnal, and volant, it is difficult to directly observe their feeding habits, which has resulted in their classification into broadly defined dietary guilds (e.g., insectivores, carnivores, and frugivores). Apart from these broad guilds, we lack detailed dietary information for many species and therefore have only a limited understanding of interaction networks linking bats and their diet items. In this study, we used DNA metabarcoding of plants, arthropods, and vertebrates to investigate the diets of 25 bat species from the tropical dry forests of Lamanai, Belize. Our results report some of the first detection of diet items for the focal bat taxa, adding rich and novel natural history information to the field of bat ecology. This study represents a comprehensive first effort to apply DNA metabarcoding to bat diets at Lamanai and provides a useful methodological framework for future studies testing hypotheses about coexistence and niche differentiation in the context of modern high‐throughput molecular data.

## Graphical abstract

Inferring the exact diets of small, nocturnal mammals such as bats is notoriously difficult. Limited knowledge of what bats actually eat leaves important trophic interactions and food web networks unclear. Using molecular barcoding of plants, vertebrates, and invertebrates, we report fine‐scale dietary detection for 25 species of Belizean bats.

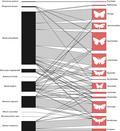

## 1 INTRODUCTION

High‐throughput sequencing (HTS) has enabled novel insights into animal diets (e.g., De Barba et al., 2014; Shehzad et al., 2012). Prior to the advent of HTS, most investigations into animal diets relied on bulk fecal sorting and identification (e.g., Howell & Burch, 1973; Lopez & Vaughan, 2007; Medellin, 1988). Bulk sorting is contingent upon the ability to identify plants, vertebrates, and insects from fragments, which requires substantial taxonomic expertise and time. In addition, the utility of this approach is limited when the most taxonomically informative portions of the diet item are not consumed (e.g., the elytra of beetles; Czaplewski et al., 2018). Recently developed molecular approaches known collectively as “DNA metabarcoding” have revolutionized investigations into animal ecology by providing methods that are fast, scalable, and customizable to particular dietary taxa of interest (De Barba et al., 2014; Tab erlet et al., 2007b; Riaz et al., 2011). DNA metabarcoding leverages universal primers to target many consumed taxa at once and is particularly useful for studying the diets of animals with elusive lifestyles that prevent the use of more traditional methods such as direct observation. For example, this approach has been used over the last decade to profile the diets of species ranging from small, cryptic mammals such as voles (Soininen et al., 2009) to rare, highly vulnerable carnivores (Hacker et al., 2021; Havmøller et al., 2020). DNA metabarcoding therefore has the potential to both inform basic natural history and ecology for many mammals and elucidate key dietary requirements for others of conservation concern.

One group of mammals that greatly benefits from the increased dietary resolution of metabarcoding is the bats (Order: Chiroptera). Bats are a taxonomically and ecologically diverse clade of mammals, and yet, the detailed dietary ecology and natural history of many species remain poorly described (Simmons, 2005). The feeding habits of bats are particularly difficult to directly observe because of their small body sizes, nocturnal foraging activity, habitat, and ability to fly (Kunz & Fenton, 2003; Simmons & Conway, 2003). Our understanding of the dietary ecology of this hyper‐diverse clade has thus changed slowly since historical descriptions broadly classifying bats as insectivorous, frugivorous, nectarivorous, omnivorous, carnivorous, sanguivorous, or piscivorous (Allen, 1939; Gardner, 1977). In the intervening decades, ecologists and evolutionary biologists have further extended knowledge of dietary ecology for many species by studying aspects of functional morphology (Brokaw & Smotherman, 2020; Dumont et al., 2009; Murillo‐García & De la vega, 2018; Santana et al., 2011), echolocation, and behavior (Arbour et al., 2019; Korine & Kalko, 2005), and also by bulk fecal sorting or isotopic niche analyses (García‐Estrada et al., 2012; Howell & Burch, 1973; Lopez & Vaughan, 2007; Maynard et al., 2019; Oelbaum et al., 2019). While providing important evidence for general food habits, these methods are often more agnostic to a finer‐scale niche partitioning. For example, functional morphology and behavior can discriminate between aerial‐hawking and surface‐gleaning insectivores, but these methods lack the resolution necessary to determine which species of insects different bat species are eating, or to evaluate hypotheses about how aerial insectivores avoid competition for flying insects. Even macroscopic fecal analyses that permit identification of some dietary items (e.g., insects that have identifiable hard parts, plants with very small‐seeded fruits) may miss others (e.g., soft‐bodied prey, large‐seed fruits, body fluids such as blood).

In light of these limitations, there have been many recent studies applying fecal DNA metabarcoding to the study of bat diets. Such DNA‐based techniques are commonly used to study the diets of bats in temperate areas of the world, where most species feed nearly exclusively on insects (Aizpurua et al., 2018; Galan et al., 2018; Wray et al., 2018). In contrast, this technique is less frequently used to inventory the diets of the more trophically diverse tropical bats (but see Hayward, 2013). This method has the power to fundamentally revise our understanding of feeding habits of tropical bat species. For example, DNA barcoding showed that *Glossophaga soricina*, a species traditionally assumed to be a nectar specialist owing to its specialized morphology, actively takes insects during some seasons of the year, making it functionally omnivorous (Clare et al., 2014). DNA metabarcoding has also characterized the diets of single species of tropical bats, including common vampire bats (*Desmodus rotundus*), leading to a revised understanding of prey choice in human‐altered landscapes (Bohmann et al., 2018; De Oliveira et al., 2020).

Few studies leverage the power of DNA metabarcoding to document the diets of multitrophic assemblages of bats, which often include sympatric fruit, nectar, insect, and blood‐feeding species. To address this knowledge gap, we sampled fecal material from 25 ecologically diverse species of bats from the tropical dry forests of the Lamanai Archaeological Reserve, Orange Walk District, Belize, to profile their diets using DNA metabarcoding of invertebrates, vertebrates, and plants. We also apply network theory to analyze patterns in the ecological assemblage of bats at Lamanai. Network‐based approaches provide helpful statistical tools to measure system‐wide attributes of ecological communities. For example, through the generation of null interaction network models, it is possible to test hypotheses about diet specificity in the observed community (Dormann et al., 2009). Such approaches have been used in the past to describe various ecological networks from pollinator assemblages to army ant diets (Chacoff et al., 2012; Hoenle et al., 2019), but to our knowledge have not been applied to multitrophic mammal communities. Our results give a first look into the network structure of the Lamanai bat community and provide novel, taxon‐level insights into the diets of 25 bat species, encompassing representatives of all major ecological guilds.

## 2 METHODS and MATERIALS

### 2.1 Sample collection

**FIGURE 1:**
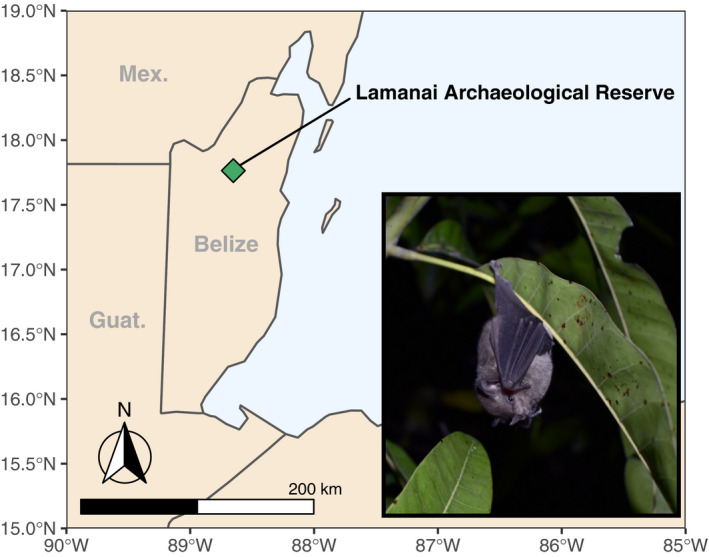
Map depicting field site location within Belize, Central America (diamond). Inset: one of the focal species of the study, *Glossophaga soricina*

We collected fecal samples from bats captured in and around the Lamanai Archaeological Reserve in Orange Walk District, Belize (17.75117°N, 88.65446°W), from April to May 2018 under Belize Forest Department Permit WL/2/1/18(16). All field protocols followed the recommendations for humane capture and handling of live mammals outlined by the American Society of Mammalogists (Sikes et al., 2016) and were approved by the American Museum of Natural History Animal Care and Use Committee (AMNH IACUC‐20180123). For a total of 13 nights, we deployed 5–10 ground‐level mist nets and 1–2 harp traps within the Lamanai Archaeological Reserve (Figure 1). Captured bats were placed into individual clean cloth holding bags. We collected fecal samples using sterilized forceps directly from the bottom of holding bags. Each sample was placed into a sterile‐barcoded tube and immediately submerged in liquid nitrogen. Between uses, holding bags were washed in an industrial laundry to minimize cross‐contamination between sampling sessions. Forceps were twice sterilized between uses with DNA‐Away solution (Molecular Bioproducts, Inc., San Diego, CA) and water. In total, we collected 80 guano samples from 25 species (Table 1). Samples were shipped frozen to the American Museum of Natural History (AMNH) and stored at −80°C prior to DNA extraction.

**TABLE 1:** Species sampled, including number of individuals and their dietary assessments in the literature and as a result of this study

Family	Taxon	*n* (65)	A priori Diet (Allen, 1939; Gardner, 1977)	Empirical Diet (this study)
Emballonuridae	*Rhynchonycteris naso*	4	Insects	Insects
Mormoopidae	*Mormoops megalophylla*	1	Insects	Insects, Plants
	*Pteronotus mesoamericanus*	4	Insects	Insects, Plants
Molossidae	*Molossus nigricans*	8	Insects	Insects
Natalidae	*Natalus mexicanus*	2	Insects	Insects
Phyllostomidae	*Artibeus intermedius*	1	Fruit, likely also insects	Mostly fruit with some insects
	*Artibeus jamaicensis*	3	Fruit, Insects	Mostly fruit with some insects
	*Artibeus lituratus*	5	Fruit, Insects	Mostly fruit with some insects
	*Dermanura phaeotis*	1	Fruit	Fruit
	*Dermanura watsoni*	3	Fruit	Fruit
	*Carollia perspicillata*	2	Fruit, Flowers, Insects	Fruit, Insects
	*Carollia sowelli*	10	Fruit, Insects	Mostly fruit
	*Chrotopterus auritus*	1	Insects, Vertebrates, possibly fruit	Insects, Fruit
	*Gardnernycteris keenani*	1	Insects	Insects
	*Glossophaga soricina*	3	Fruit, Insects, Nectar, Pollen	Mostly fruit, nectar, or pollen
	*Lophostoma evotis*	2	Insects, possibly fruit	Mostly plant material, some insects
	*Mimon cozumelae*	1	Insects, Vertebrates, maybe plants	Insects, Plants
	*Sturnira parvidens*	3	Fruit	Fruit
	*Trachops cirrhosus*	1	Insects, Vertebrates, possibly some fruit	Insects, Arachnids, Plants
Vespertilionidae	*Bauerus dubiaquercus*	1	Insects	Insects, Plants
	*Eptesicus furinalis*	2	Insects	Insects
	*Myotis elegans*	2	Insects	Insects
	*Myotis pilosatibialis*	1	Insects	Insects
	*Rhogeessa aeneus*	2	Insects	Insects

### 2.2 DNA extraction

For each sample, we performed extractions using several fecal pellets (up to 0.25 g total). We extracted total DNA using the QIAamp PowerFecal DNA Kit (MO BIO Laboratories, Qiagen Co.) following the manufacturer's instructions with the following alterations; prior to homogenization, we incubated fecal samples in the provided lysis solutions for 10 min at 70°C. Next, we disrupted the fecal material in a Fisherbrand Bead Mill 24 Homogenizer (Fisher Scientific) at 6 m/s for 1–2 min, until the fecal slurry was fully homogenized. At the elution step, we eluted with 55°C PCR‐grade water and incubated columns for two minutes prior to centrifugation. In addition to our samples, we extracted one “blank” water sample per extraction kit. Purified DNA extracts were preserved at −25°C prior to library preparation.

### 2.3 Metabarcode library preparation

We amplified plant, invertebrate, and vertebrate DNA using three sets of previously developed universal primers (Table 2). We targeted the P6 loop of the chloroplast *trnL* gene for plants (Taberlet et al., 2007) and the mitochondrial 16S mtDNA for invertebrates (De Barba et al., 2014). For the four bat species known or suspected to eat vertebrates—*Noctilio leporinus, Trachops cirrhosus, Chrotopterus auritus,* and *Mimon cozumelae*—we also used a universal vertebrate primer targeting the 12S‐V5 mitochondrial region (Table 2; Riaz et al., 2011). Though short, these mini‐barcodes have been shown to be both taxonomically discriminatory and low in bias and have undergone extensive validation for the specific purpose of fecal metabarcoding. Because both the 16S and 12S universal primers co‐amplify host DNA, we used a mammal‐specific blocking primer in all master mixes targeting 16S (De Barba et al., 2014), and custom host‐specific blocking primers for the four bat fecal extracts amplified with 12S universal primers (Table 2). Blocking primers have high specificity to host sequences but contain a 3′‐end C3 modifier that prevents amplification, leading to decreased representation of host sequences by the final round of PCR (Vestheim & Jarman, 2008). Briefly, we used the software *ecoPrimers* (Riaz et al., 2011) to design the host‐specific 12S blocking primers from publicly available bat 12S sequences on NCBI GenBank. Following in silico blocking primer design, we checked the alignments of each primer with host 12S sequences and 12S sequences from hypothesized prey taxa, ensuring that there would be a sufficient number of 3′ mismatches to prevent coblocking of prey DNA. All nonblocking primer constructs contained Illumina adapters for use with the Nextera XT Index Kit (Illumina Inc.). We amplified each target region in separate 20 μl reactions per fecal sample containing 1× Kapa HiFi HotStart ReadyMix, 1× of each primer, 10× of blocking primer, and 2 μl DNA extract (De Barba et al., 2014; Smith & Peay, 2014). Cycling protocols for all primers followed DeBarba et al. (2014): an initial denaturation step of 15 min at 95°C, followed by 55 cycles of 30 s at 94°C, 90 s at 55°C, and no elongation step (De Barba et al., 2014). Three PCR‐negative controls (one per amplicon plate) were performed to check for contamination and were pooled together prior to indexing. Amplicons, including extraction and PCR‐negative controls, were checked on a 2% agarose gel prior to indexing to confirm amplification of target fragments and check for contamination. At no point was a visible band detected for any negatives, indicating low amounts of ambient contamination.

**TABLE 2:** List of primers used in this study

Target Taxon	Target Gene	Primer Name	Primer Type	Primer Sequence 5′–3′	Reference	Prod. Length (bp)
Invertebrates	16S mtDNA	16S‐MAVF	Fwd	CCAACATCGAGGTCRYAA	De Barba et al. (2014)	36
		16S‐MAVR	Rev	ARTTACYNTAGGGATAACAG	De Barba et al. (2014)	
		16S‐MAVBl	Block	CCTAGGGATAACAGCGCAATCCTATT‐C3	De Barba et al. (2014)	
Plants	trnL	g	Fwd	GGGCAATCCTGAGCCAA	Taberlet et al. (2007)	51
		h	Rev	CCATTGAGTCTCTGCACCTATC	Taberlet et al. (2007)	
Vertebrates	12S‐mtDNA	12SV5‐F	Fwd	TTAGATACCCCACTATGC	Riaz et al. (2011)	98
		12SV5‐R	Rev	TAGAACAGGCTCCTCTAG	Riaz et al. (2011)	
		HomoB	Block	CTATGCTTAGCCCTAAACCTCAA﻿CAGTTAAATCAACAAAACTGCT‐C3	De Barba et al. (2014)	
		12SF‐MimBlk	Block	CTATGCTTAGCCTTAAACCTAGAAAATTATTATAAC‐C3	This study	
		12SF‐ChrotBlk	Block	ATGCTTAGCCGTAAACTTAAAGAATTTGCTAAAA‐C3	This study	
		12SF‐TrachBlk	Block	CCCACTATGCCTAGCCTTAAACCTAAAGAAT‐C3	This study	
		12SF‐NoctBlk	Block	CTATGCTTAGCCATAAACCTAAAAA‐C3	This study	

Following the initial amplicon PCR, we cleaned and size‐selected each PCR using AMPure XP beads at a ratio of 1.8–2.0×, which retains fragments of approximately 100 bp or larger (Agencourt Biosciences). Purified amplicons were indexed using the Nextera XT series of barcodes in an 8‐cycle PCR following the manufacturer's recommendations (Illumina Inc). Indexed amplicons were cleaned with AMPure XP beads prior to normalization, and fragment size was checked on a Bioanalyzer DNA High Sensitivity Chip (Agilent Technologies). Finally, we normalized all amplicons to a final concentration of 4.5 nM and pooled aliquots of each sample. Amplicons were sequenced on Illumina MiSeq platform (Illumina) at the Bioinformatics and Computational Genomics Laboratory at the City University of New York using v2 2 × 150 bp chemistry and a 20% PhiX spike‐in.

### 2.4 Molecular OTU analysis

Raw data were first preprocessed and demultiplexed with the MiSeq Reporter Generate FASTQ workflow (Illumina). Primer sequences were trimmed from forward and reverse sequence reads using Cutadapt v. 1.4.2 (Martin, 2011). Next, we used OBITools v. 1.01 (Boyer et al., 2016) to quality‐filter, join, and taxonomically annotate paired‐end reads. Briefly, we first constructed reference databases for the 16S, 12S, and *trnL* genes using the in silico ecoPCR tool and the EMBL 141 release (Boyer et al., 2016; Kanz et al., 2005). Next, we aligned and joined paired‐end reads using the *illuminapairedend* command, and filtered the dataset of any sequences that could not be successfully aligned (mode! = “joined”). This step resulted in a total of 2,476,777 aligned reads across the full dataset. Reads were further dereplicated using the *obiuniq* command and denoised by retaining only those sequences that were between 30 and 150 bp in length and had a count >2 in the dataset. Finally, we cleaned the sequences of PCR errors using the *obiclean* command, specifying to keep only those sequences with no variants with a count greater than 5% of their own count (−r 0.05 option). This step left a total of 24,584 molecular operational taxonomic units (MOTUs), or nonvariant sequences, for downstream taxonomic classification. Using the *ecotag* command, we assigned taxonomy to the dataset in three separate steps (plants, invertebrates, and vertebrates) and tabulated the 90% matching MOTU hits. After filtering all MOTUs that were not classified at least to the taxonomic level of Order, we were left with a total of 824 MOTUs encompassing invertebrates and plants; there were no hits assigned to vertebrates. Finally, we manually curated the MOTUs and removed only a few spurious hits (e.g., marine decapods) and any 12S sequences identified as host DNA. The minimum identity match for the dataset was 90.2%, while the highest was 100%. The mean match identity was 97.1%, indicating a well‐annotated final dataset.

All preprocessing steps were carried out in R version 4.0 using the following packages: *phyloseq* v. 1.32.0 (McMurdie & Holmes, 2013), *vegan* v 2.5.6 (Oksanen et al., 2017), *microbiome* v 1.10.0 (Lahti et al., 2017‐2020), *microbiomeSeq* (Ssekagiri et al., 2017), and *decontam* v. 3.8 (Davis et al., 2018) (R Development Core Team, 2016). We began by filtering the dataset of potential contaminants identified in negative control using the “frequency” method implemented in *decontam*. This step eliminated the extraction negative control sample. Next, we further filtered the dataset to retain only samples with a minimum of four unique observed MOTUs, resulting in a total dataset of 65 samples. To account for differences in library size, we scaled the dataset using the “log10 + 1” method (Lahti et al., 2017‐2020) and used this transformed feature table for all subsequent analyses. To test for differences in MOTU composition among guilds, we performed nonmetric multidimensional scaling (NMDS) on the Bray–Curtis and Jaccard distance matrices. We performed permutational ANOVA (PERMANOVA) on both distances to account for differences both weighted by abundance (Bray–Curtis) and due to unweighted presence/absence (Jaccard). Next, we used the R package *bipartite* v 2.15 (Dormann et al., 2014) to visualize the dietary network and to calculate and create adjacency plots. Finally, we aggregated observations together by bat species and computed dietary specialization indices by calculating both the Shannon diversity index (computed at the MOTU level) and the H′2 specialization index for each species (Blüthgen et al., 2006; Shannon, 1948). To test whether dietary specialization in our observed network differs from expectations under a null model, we generated 100 null networks using the “vaznull” method, which randomizes links while preserving the connectance structure of the observed network (Vázquez et al., 2007). We compared the mean Shannon diversity and H′2 values of our community with those of the nulls using a one‐sample *t* test after checking the null estimates for normality. To assess within‐species variation for species with multiple observations, we computed the local contribution to beta diversity (LCBD; Legendre & De Cáceres, 2013; Ssekagiri et al., 2017), which considers the uniqueness of each sample to the overall variation in community composition for each group.

## 3 RESULTS

### 3.1 Community‐level dietary attributes

**FIGURE 2:**
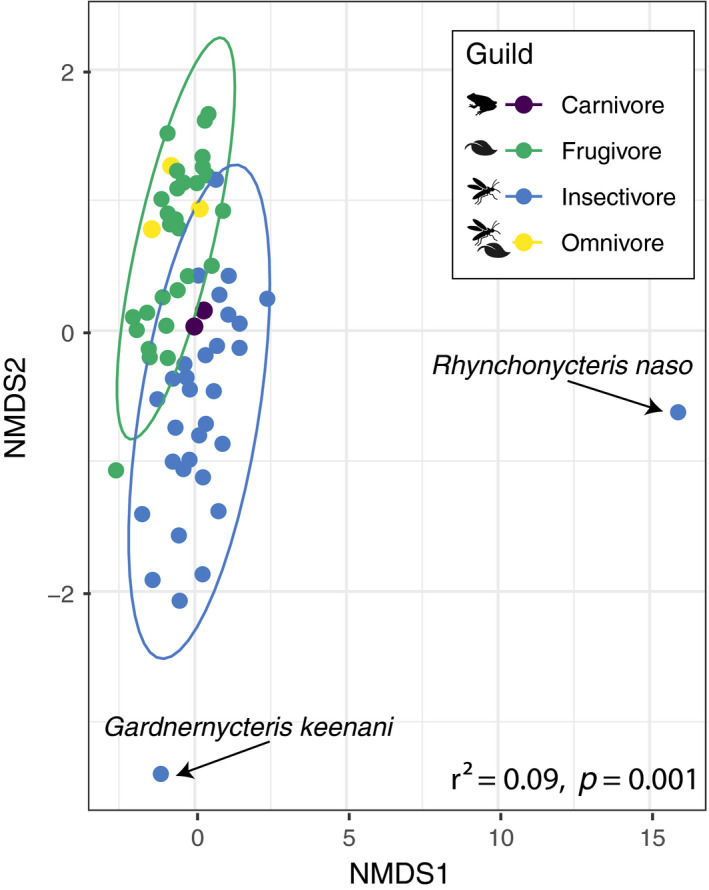
NMDS ordination of molecular bat diets from Lamanai. Each dot represents an individual fecal sample. Points are colored according to dietary guild as shown in Table 1. Lower right depicts PERMANOVA results of host guild on the Bray–Curtis distance matrix

Of the 80 collected samples, 65 passed quality‐filtering steps and were retained in the final analyses. In the quality‐filtered dataset, we recovered 811 dietary MOTUs split across both invertebrate (617) and plant (194) taxonomic orders. We did not identify any nonhost vertebrates in the final dataset. The average number of MOTUs per sample was 2,630 (range: 7–94,791). Of the top 25 overall most frequent taxa, the most frequently observed invertebrate orders were the Hemiptera, Coleoptera, Lepidoptera, and Diptera. The most common plant orders were the Rosales, Piperales, and Sapindales. We used nonmetric multidimensional scaling (NMDS) to visualize the separation among traditional dietary guilds in the community (Figure 1). Using PERMANOVA, we found that traditional dietary guilds (Bray–Curtis: *F*_3,64_ = 2.38, *r* ^2^ = 0.09, *p* = 0.001; Jaccard: *F*_3,64_ = 1.86, *r* ^2^ = 0.08, *p* = 0.001) and individual species (Bray–Curtis: *F*_20,64_ = 1.54, *r* ^2^ = 0.39, *p* = 0.001; Jaccard: *F*_20,64_ = 1.36, *r* ^2^ = 0.37, *p* = 0.001) had significantly different dietary compositions. Because PERMANOVA can sometimes be affected by nonhomogeneity of dispersion for unbalanced sampling schemes (Anderson & Walsh, 2013), we also performed a permutational dispersion test, which was significant (*F*_3,61_ = 46.19, Nperm = 999, *p* = 0.001). However, upon visual inspection of the ordination we determined that intragroup dispersion alone was not likely to be driving the differences between feeding guilds, due to the presence of only two outlier species skewing within‐group heterogeneity of dispersion (Figure 2).

**FIGURE 3:**
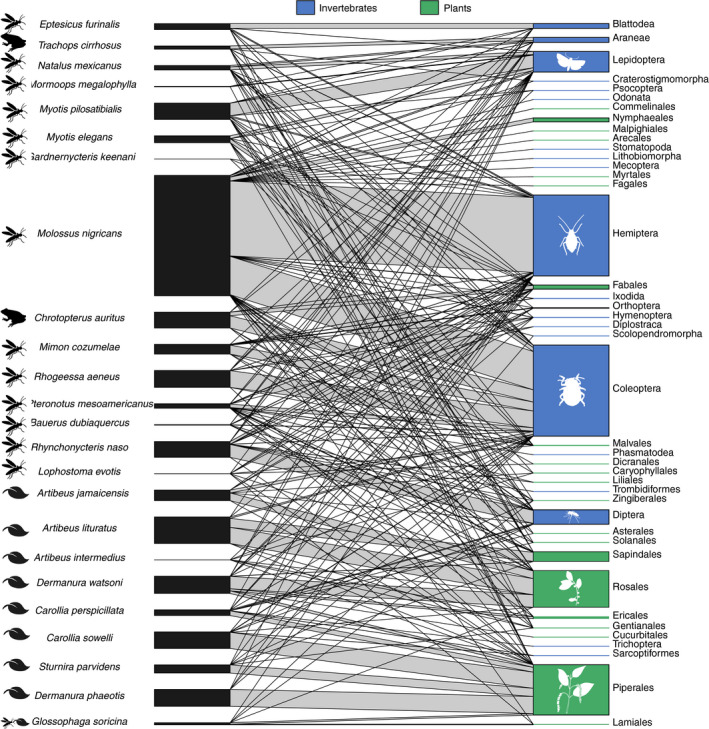
Bipartite network showing associations between bats (left) and invertebrate and plant diet items summarized at the level of taxonomic Order (right). Connecting bar width represents the frequency of observation between each bat and each dietary item

We constructed bipartite networks to visualize dietary connections between each bat species and the invertebrate and plant dietary components summarized to taxonomic Order (Figure 3). The community was characterized overall by a high number of infrequent connections. A few of the most frequent associations in the community were between *Molossus nigricans* and both beetles (Coleoptera) and true bugs (Hemiptera). Coleoptera were also frequently associated with *C. auritus*, *M. cozumelae*, and *Rhogeessa aeneus*. True flies (Diptera) were most often associated with the small periaquatic insectivore *Rhynchonycteris naso*, while moths and butterflies (Lepidoptera) showed many associations with the insectivores *Myotis elegans*, *Myotis pilosatibialis*, and *Natalus mexicanus*. The spiders (Araneae) and roaches (Blattodea) were nearly exclusively associated with *T. cirrhosus* and *Eptesicus furinalis*, respectively.

Plant dietary components were classified into a smaller number of taxonomic orders (Figure 3). All species of fig‐eating bats in the genus *Artibeus* showed frequent associations with the Rosales (the order that contains fig trees and fruits of *Cecropia* spp.), but we also detected plants of the order Sapindales, a diverse order that includes citrus fruits, mangos, and mahogany, in the diet of *Artibeus lituratus*. Plants of the Piperales were often detected in the diets of *Carollia sowelli, Carollia perspicillata, Sturnira parvidens*, and the omnivorous *G. soricina*. The two species of the genus *Dermanura* had frequent interactions with both Piperales and Rosales.

**FIGURE 4:**
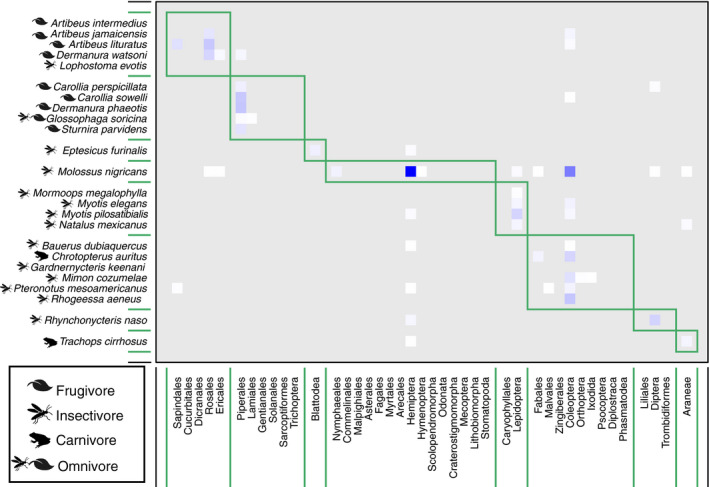
Adjacency matrix summarizing community subgroups according to detected dietary items. Bat species are shown along the vertical axis, and plant and invertebrate Orders are shown along the horizontal axis. Boxes demarcate groupings, and darker blue squares represent more frequent associations

Given the complexity of the bipartite interactions, we generated an adjacency matrix to visualize possible modules within this community. The adjacency matrix defined a total of eight modules in the community (Figure 4). Two models contained all of the primarily fruit‐feeding bats, while other modules contained all of the insectivorous or carnivorous species. Four modules contained only a single taxon, highlighting the unique food items detected for these four species—*E. furinalis, M. nigricans, R. naso,* and *T. cirrhosus*.

The bat community observed at Lamanai was significantly different in both specialization (H′2_observed_ = 0.765, *t* = −5520.4, *df* = 99, *p*‐value < 0.001) and Shannon diversity (Shan_observed_ = 3.05, *t* = 1,439.8, *df* = 99, *p*‐value < 0.001) compared with 100 null bipartite networks.

### 3.2 Fine‐scale associations between bats and insects

**FIGURE 5:**
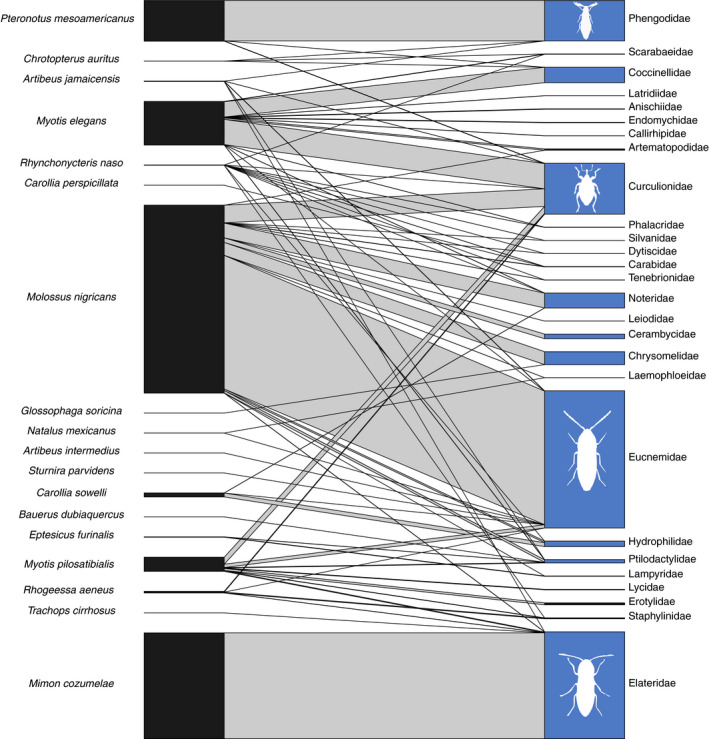
Bipartite network for the beetles (Coleoptera). Connecting bar width represents the frequency of observation between each bat (left) and each dietary item (right)

Order‐level diet information is known for many insectivorous bat species, but finer taxonomic information about prey insects is often lacking. We therefore also described the associations between bats and their insect prey at the level of taxonomic Family. We created additional bipartite networks to resolve detection within the four most commonly detected groups of arthropods consumed (Coleoptera, Hemiptera, Lepidoptera, and Diptera) Within the Coleoptera, the most frequently detected associations were between just a few families (Figure 5). For example, *M. cozumelae* was often associated with the click beetles (Elateridae), while we detected a wide variety of beetle families in the feces of *M. nigricans,* most notably the false click beetles (Eucnemidae) and the weevils (Curculionidae). *Pteronotus mesoamericanus* was frequently associated with the glow worm beetles (Phengodidae). Other invertebrate families identified as components of bat diets at Lamanai were the Coccinellidae, Noteridae, Cerambycidae, Chrysomelidae, Hydrophilidae, and Ptilodactylidae.

**FIGURE 6:**
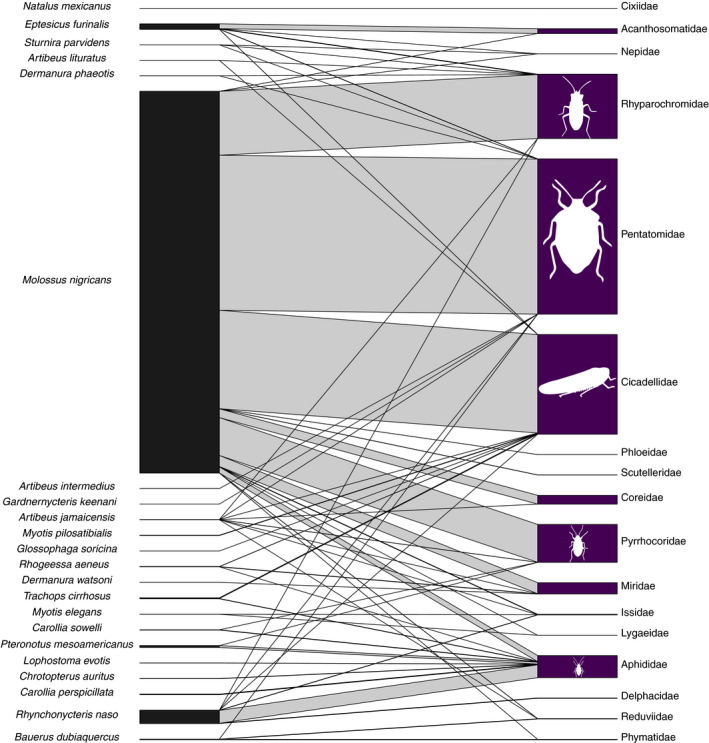
Bipartite network for the true bugs (Hemiptera). Connecting bar width represents the frequency of observation between each bat (left) and each dietary item (right)

Dietary interactions between bat species and true bugs, the Hemiptera, were similarly dominated by a few common and many less common interactions (Figure 6). *Molossus nigricans* was most often associated with stink bugs (Pentatomidae), leafhoppers (Cicadellidae), seed bugs (Rhyparochromidae), and red bugs (Pyrrhocoridae). *Eptesicus furinalis* was commonly associated with the shield bugs (Acanthosomatidae). The family Aphididae, which contains both flying and flightless aphids, was detected in the feces of many aerial insectivores, but most notably *R. naso*.

**FIGURE 7:**
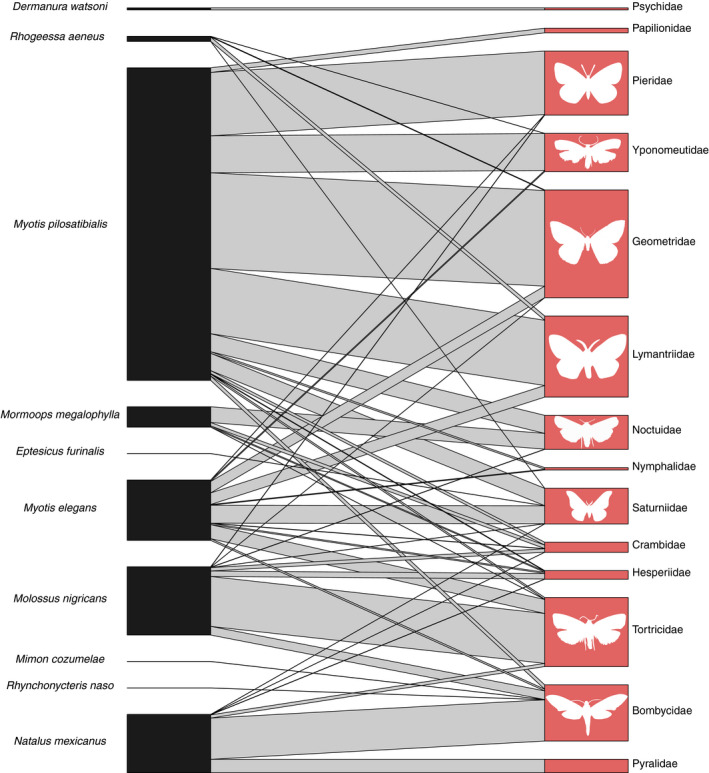
Bipartite network for the moths (Lepidoptera). Connecting bar width represents the frequency of observation between each bat (left) and each dietary item (right)

Moths and butterflies (Lepidoptera) are common prey of aerial‐hawking bats and, as expected, constituted a common component of the diets of insectivorous species at Lamanai (Sierro & Arlettaz, 1997; Figure 7). *Myotis pilosatibialis* had many associations with this insect order, in particular with the pierid (Pieridae), ermine (Yponomeutidae), geometer (Geometridae), tussock (Lymantriidae), and owlet moths (Noctuidae). *Myotis elegans* overlapped with *M. pilosatibialis* in feeding on most of these families, but was not associated with Lymantriidae, and was instead associated with Tortricidae and Saturniidae, whereas *M. pilosatibialis* was not. The other small vespertilionid in our dataset, *R. aeneus*, overlapped with both *Myotis* species in dietary resources. We did not detect moths very often in the feces of *E. furinalis*, showing only a weak association with Saturniidae. We detected noctuid and crambid moths in the feces of *Mormoops megalophylla*, and few interactions with skippers (Hesperiidae) and tortrix moths (Tortricidae). The only member of the bat family Natalidae in our dataset, *N. mexicanus*, was distinct in its strong associations with silk moths (Bombycidae) and snout moths (Pyralidae), only overlapping with other bat species in less frequent interactions. A diverse assemblage of moths were detected in *M. nigricans* feces, which overlapped with identified diet items for many other bat species. The only association of family Psychidae, the bagworm moths, was with a frugivorous bat, *Dermanura watsoni*.

**FIGURE 8:**
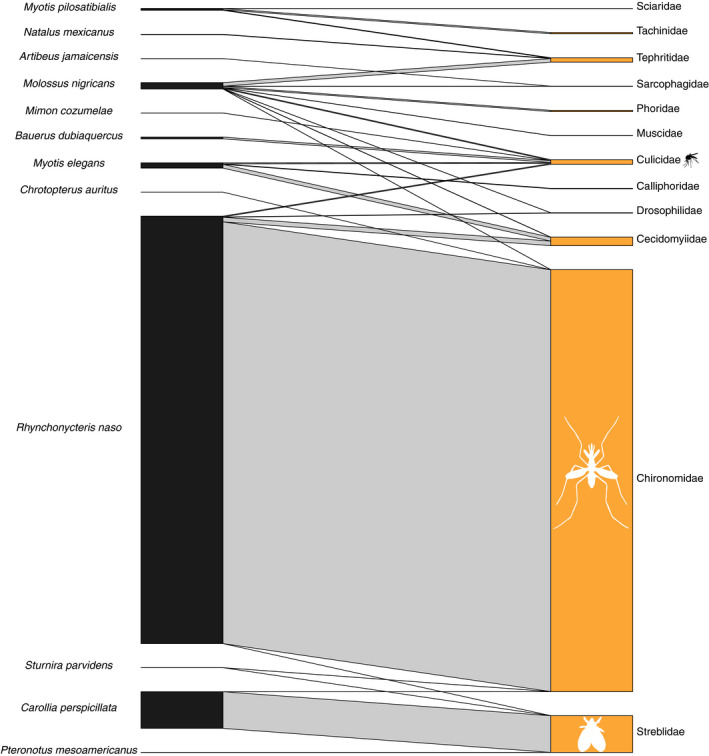
Bipartite network for the flies (Diptera). Connecting bar width represents the frequency of observation between each bat (left) and each dietary item (right)

Finally, we summarized interactions between bat species and various families of true flies (Diptera; Figure 8). Diptera were most frequently observed in the feces of *R. naso*, particularly nonbiting midges of the family Chironomidae. Other common fly families in the diets of Lamanai bats were the gall gnats (Cecidomyiidae), mosquitoes (Culicidae), and the fruit flies (Tephritidae). *Rhynchonycteris naso*, *S. parvidens*, *P. mesoamericanus*, and *C. perspicillata* feces were positive for the DNA of obligate ectoparasites of the family Streblidae, in particular members of the genus *Trichobius*.

### 3.3 Within‐species variation

**FIGURE 9:**
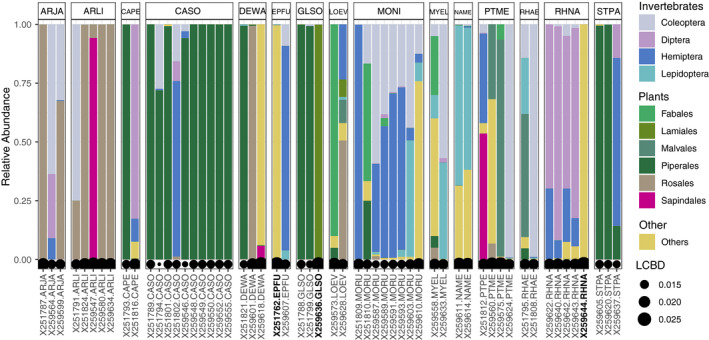
Intraspecific variation in diet for species with individual replicates (*n* = 15). Facets are organized by bat species, and each bar is an individual bat. Black circles indicate local contribution to beta diversity (LCBD) value for each individual relative to the rest of the group. Sample names in bold indicate significantly different LCBD at *p <*.*05*. Species codes: ARJA—*Artibeus jamaicensis*, ARLI—*Artibeus lituratus,* CAPE—*Carollia perspicillata,* CASO—*Carollia sowelli*, DEWA—*Dermanura watsoni*, EPFU—*Eptesicus furinalis*, GLSO—*Glossophaga soricina*, LOEV—*Lophostoma evotis*, MONI—*Molossus nigricans*, MYEL—*Myotis elegans*, NAME—*Natalus mexicanus*, PTME—*Pteronotus mesoamericanus*, RHAE—*Rhogeessa aeneus,* RHNA—*Rhynchonycteris naso*, STPA—*Sturnira parvidens*

We assessed within‐species variation in diet detection for the 15 bat species for which we had sampled multiple individuals. We found that the relative abundance of the top 10 MOTUs was fairly consistent across samples from within the same species. In fact, of more than 50 samples, only three individuals were determined to be significantly different in dietary beta diversity compared with their conspecifics (Figure 9). These three individuals belonged to the species *E. furinalis, G. soricina,* and *R. naso*.

## 4 DISCUSSION

Using DNA metabarcoding, we created novel fine‐scale dietary information for 25 species of bats from Lamanai, Belize. Overall, our results largely support traditional guild assignments, with frugivores and insectivores being readily distinguishable in beta diversity (Figure 1). However, we also identified cases with substantial guild overlap as reported in previous stable isotope analyses of the same community (Oelbaum et al., 2019). For example, the carnivorous bats were contained completely within the ellipse area of the insectivores, likely because these species—*C. auritus, T. cirrhosus,* and *M. cozumelae*—are opportunistic gleaning predators that readily take insects (Whitaker & Findley, 1980). Several frugivorous species also overlapped with the insectivore ordination space, which is consistent with previous reports that some of these species, in particular *C. sowelli* and *C. perspicillata*, consume insects during some parts of the year (Bonaccorso et al., 2007; Herbst, 1986). The only species we classified as omnivorous (based on Clare et al., 2014), *G. soricina*, nested within the frugivore guild ellipse area, suggesting that during late April–early May at this site, *G. soricina* may feed primarily on plants. Previously, *G. soricina* has been shown to take insects (Clare et al., 2014), but it is likely that populations of these bats vary considerably in their level of insectivory between seasons and across their geographic range.

Using bipartite networks and visualization of the resulting adjacency matrix, we found that the bats of Lamanai, Belize, are broadly divisible into primarily plant‐feeding or arthropod‐feeding guilds (Figures 2,3). The major split within plant‐feeding bats was driven mostly by the frequency of association with the Rosales versus Piperales. The “Rosales” module contained members of the genus *Artibeus*, which is consistent with previous reports that these bats specialize on the fruits of *Cecropia* (Rosales: Urticaceae) and *Ficus* (Rosales: Moraceae) species (Morrison, 1978; Lopez & Vaughan, 2007). Our dataset only contained two individuals of this species, so we acknowledge that additional replicates might change this module assignment. *Lophostoma* species have traditionally been considered to be insectivorous (Table 1). Nevertheless, there are anecdotal reports of the entire stomach contents of some individuals consisting of pollen or plant material during April, suggesting that these bats may be seasonally omnivorous (Goodwin & Greenhall, 1961; Howell & Burch, 1973). Longitudinal dietary analyses are needed to confirm whether *Lophostoma evotis* often uses plants as a food resource, or whether the identification of plant DNA results from trophic carry‐up from its insect prey. The other plant‐feeding group was discriminated primarily by many interactions with the Piperales and Solanales and contained bat species well known to feed on *Piper* and *Solanum* fruits, such as *C. sowelli*, *C. perspicillata*, and *S. parvidens* (Howell & Burch, 1973). Overall, species assignments in the two plant‐feeding modules are similar to those previously recovered for the Lamanai bat community using isotopic methods (Oelbaum et al., 2019).

Taxonomic information about the insect prey of tropical bats remains lacking (but see Emrich et al., 2014). Our adjacency analysis suggests the presence of six groups among the insectivorous and carnivorous bats of Lamanai, Belize (Figure 4). Four of these groups were composed of single taxa—*E. furinalis*, *R. naso, M. nigricans,* and *T. cirrhosus*. The diet of *E. furinalis* is poorly known, but there is evidence that they feed primarily on hemipterans, coleopterans, and lepidopterans (Aguiar & Antonini, 2008). In our study, *E. furinalis* was unique among the species sampled due to its association with Blattodea (Figures 3,4). *Rhynchonycteris naso* was uniquely associated with chironomid midges, which were more frequently detected compared with those of other bat species (Figure 8). Previous isotopic characterizations of *R. naso* diet suggest that these bats occupy a key niche within Belizean bat communities (Oelbaum et al., 2019), likely the result of this species' unique roosting and foraging ecology. *R. naso* can typically be found roosting on exposed surfaces overhanging bodies of water (Fenton et al., 2001) and forage more extensively on small aquatic insects than other aerial insectivores (Becker et al., 2018). In another case of ecologically specialized morphology and behavior, the large body size and high aspect ratio wings of *M. nigricans* suggest that they forage at great height in open areas, giving them access to different insects not likely to be consumed by bats foraging in cluttered habitat (Aldridge and Rautenbach, 1987). We found *M. nigricans* to have a diverse diet and detected the highest quantity and diversity of arthropods of any studied bat species in the Lamanai community (Figure 2). However, we detected a significant relationship between the number of sampled individuals and the number of MOTUs for each species, so we suspect that further sampling of this community is needed to determine large‐scale patterns in dietary diversity (Alberdi et al., 2019; Zinger et al., 2019).

Finally, *T. cirrhosus* was also found to be unique in its dietary associations at Lamanai; this was principally driven by the detection of spiders, notably the banana spiders (Trechaleidae: *Cupiennius*) and the crab spiders (Thomisidae) (Figure 2). However, we also only captured a single *Trachops* individual, so we caution against overinterpreting these results. *Trachops cirrhosus* has previously been reported to eat arachnids (Bonato et al., 2004; Leal et al., 2018), though they are well known for eating frogs (Ryan & Tuttle, 1983; Tuttle et al., 1982). Our data are consistent with a previous report of *T. cirrhosus* feeding at a high trophic level as indicated by δ^15^N isotopes (Oelbaum et al., 2019). Spiders are known to be enriched in δ^15^N compared with other arthropods (Girard et al., 2011), suggesting that stable isotopes may be unable to discriminate between vertebrate prey and nitrogen‐enriched arthropods in the diets of some species. We did not identify any vertebrate prey in the feces of the carnivorous bats in our dataset, and considering the specificity of our host‐blocking primers and the fact that host 12S DNA did amplify, we interpret this result as a lack of detection of vertebrate prey rather than an instance of off‐target blocking. A potential drawback of metabarcoding is that it provides a snapshot in time, where species not consumed on the night of sampling will not be detected in the diet. It is therefore possible that carnivorous species simply did not consume vertebrates on the night we sampled them. Alternatively, the elevated nitrogen ratios of carnivorous bats such as *T. cirrhosus* reported in Oelbaum et al. (2019) may have been at least in part the result of nitrogen enrichment in arachnid prey. These data suggest that molecular surveys of carnivorous bat diets are needed to determine the frequency of vertebrate prey in the diet, whether there is significant variation from night to night and/or between individual bats in what prey is taken, and whether their prey preferences vary across the year in this strongly seasonal environment. We postulate that during late April– early May, the dry season in Belize, the availability of frogs and other periaquatic vertebrate prey may be limited.

The last two groups of the community were somewhat consistent with the division between aerial hawkers and surface gleaners (Figure 4). One group contained the species *M. megalophylla, M. elegans, M. pilosatibialis,* and *N. mexicanus*. This grouping is supported by the high number of observations of Lepidoptera, consistent with previous reports of moths and beetles in the fecal material of these animals (Table 1; Rolfe et al., 2014; Torres‐Flores & López‐Wilchis, 2019; Whitaker & Findley, 1980). The other multispecies insectivore group contained *Baeurus dubiquercus, C. auritus, Gardnernycteris keenani, M. cozumelae, P. mesoamericanus,* and *R. aeneus*. This grouping combined bat species that fall across the aerial hawker (e.g., *P. mesoamericanus* and *R. aeneus*) and surface gleaner (e.g., *M. cozumelae, C. auritus*) modes. However, a previous study found the isotopic niche spaces of *M. cozumelae* and *C. auritus* overlapped completely (Oelbaum et al., 2019), and a previous study of the diet of *G. keenani* has also reported large beetle fragments, further supporting this module (Whitaker & Findley, 1980). Likewise, beetles have been demonstrated to be important parts of the diets of various *Pteronotus* species (Rolfe et al., 2014; Salinas‐Ramos et al., 2015), although it has been reported that *P. mesoamericanus* in Costa Rica feeds mostly on Lepidoptera and Diptera (de Oliveira et al., 2020). The diet of *R. aeneus* remains poorly known, but our results suggest these bats may prey on moths in the family Lymantriidae (tussock moths) and flying beetles of the Order Staphylinidae (Figure 5).

Overall, our groupings are broadly consistent with prior data on the diets of bat species (Leal et al., 2018; Lopez & Vaughan, 2007; Sánchez & Giannini, 2018; Whitaker & Findley, 1980); one notable exception is that our data show that many insectivorous species have associations with plants. While this may be due to detection of plant material “carried up” through arthropod prey, many of the bat species in our dataset have historical reports of pollen or seeds in fecal material. For example, up to 13% of the fecal mass of *M. pilosatibialis* and 12% of that of *T. cirrhosus* collected between 1972 and 1974 in Panama and Costa Rica was composed of unidentified seeds (Whitaker & Findley, 1980), similar to the few associations we detected between these species and plants in our study (Figure 3). In a similar set of cases, we also detected associations between frugivorous bats and several families of arthropods (Figures 5, 6, 7, 8). Some of these associations may be explained by facultative insectivory, cases in which bats morphologically and/or behaviorally specialized for frugivory may opportunistically take insects (Clare et al., 2014; Herrera et al., 2002; Lopez & Vaughan, 2007). *Artibeus* and *Dermanura* species have been shown to obtain their nutrition nearly exclusively from plant material regardless of season (Herrera et al., 2002), yet we detected associations between them and several families of arthropods (Figures 5, 6, 7, 8). Our data support the hypothesis that members of the genera *Artibeus* and *Dermanura* are obligate frugivores, but members of these groups may occasionally or incidentally take insects. Further molecular dietary analysis can confirm whether this is the case across their geographic range and across different seasons.

An important consideration for DNA metabarcoding is that to truly know dietary niche breadth for any species requires many replicates. Because of our limited sample size (65 samples for 25 species), we do not intend to present these data as the definitive niche breadths for any species. Given that we detected variation in diet among individuals of the same species (Figure 9), we caution over interpretation of these results without further research into this system. One study on *Miniopterus schreibersii* diet estimated that more than 30 samples would be needed to capture > 90% of the MOTU diversity at a given site (Aizpurua et al., 2018). In spite of the limited number of samples, our study elucidates previously unrecognized trophic connections and serves as a roadmap for testing hypotheses about niche differentiation in cryptic tropical mammal communities. Still, we recognize that DNA metabarcoding is limited in a few key ways. First, it is not directly possible to tell whether identified diet components were directly consumed by bats or whether they were initially consumed by prey insects and only secondarily detected in bat guano. We also must acknowledge that we did not use a positive control reference library of local Belizean plants, animals, and insects to compare our MOTUs against, which limits our ability to assess the robustness of our taxonomic assignments. However, because our analyses are robust to taxonomic uncertainty (i.e., the lack of a good species‐level match moves the taxonomic assignment up a level in the hierarchy), we believe that the higher taxonomies of our results are likely to be fairly accurate. In the future, it will be necessary to expand upon this study with more samples and a curated reference for positive controls.

Another potential limitation to our approach is that it is limited to a snapshot in time. Metabarcoded feces represent, at most, a sampling of individual dietary components over a one‐ or two‐night period. In addition, primer bias is a concern that applies to any use of “universal” barcodes, as some consumed taxa may not be detected depending on which primers are used (Piñol et al., 2015). While this is a valid limitation for long‐term ecological questions, we suggest that a DNA metabarcoding approach may be more suitable than isotopic data for addressing certain ecological and evolutionary questions because of, rather than in spite of, the relatively short temporal scale captured using this method. A previous study of vampire bats in the Lamanai area failed to find links between diet inferred from stable isotopes and gut microbiome turnover (Ingala et al., 2019), but this might be because isotope data record diet averaged over a relatively long timespan. DNA metabarcoding may be the most appropriate and powerful research technique for linking diet to changes in gut microbiomes because it captures diet on a temporally comparable scale to the rate of turnover in bacterial communities (David et al., 2014; Voigt et al., 2012). Future work could explicitly test the suitability of using long‐ and short‐term diet inference methods for addressing these and other novel questions in ecology and evolution.

## 5 CONCLUSIONS

In this study, we created the first multitrophic molecular dietary inventory for a Neotropical small mammal community by leveraging the power of a DNA metabarcoding approach. Applying network theory, we found that each bat species varied considerably in their associations with invertebrate and plant groups, with no two species completely overlapping in detected food items. Our results provide necessary fine‐scale information about bat diets that can be expanded upon with more replicates to test hypotheses about niche structure and competition in tropical mammal communities. In summary, our work provides a framework for understanding ecological diversity and can be applied to other species with cryptic habits whose diets are poorly known.

## CONFLICT OF INTEREST

The authors declare no conflicts of interest.

## AUTHOR CONTRIBUTIONS

**Melissa R. Ingala:** Conceptualization (lead); Data curation (lead); Formal analysis (lead); Funding acquisition (equal); Investigation (lead); Methodology (lead); Project administration (lead); Resources (supporting); Software (lead); Visualization (lead); Writing‐original draft (lead); Writing‐review & editing (lead). **Nancy B. Simmons:** Conceptualization (supporting); Funding acquisition (lead); Supervision (equal); Writing‐original draft (equal). **Claudia Wultsch:** Conceptualization (supporting); Methodology (supporting); Supervision (supporting); Writing‐original draft (equal). **Konstantinos Krampis:** Investigation (supporting); Methodology (supporting); Writing‐original draft (equal). **Kaiya L. Provost:** Formal analysis (supporting); Writing‐original draft (equal). **Susan L. Perkins:** Conceptualization (supporting); Funding acquisition (supporting); Methodology (supporting); Resources (equal); Supervision (lead); Writing‐original draft (equal).

## Data Availability

All code generated for the analyses in this paper is available at https://github.com/MelissaIngala/BelizeBatDiets. Raw data for all metabarcode libraries are available on the NCBI Sequence Read Archive (SRA) under BioProject number PRJNA716622. MOTU table (https://doi.org/10.6084/m9.figshare.14265017), sample metadata (https://doi.org/10.6084/m9.figshare.14265050), and taxonomic classifications (https://doi.org/10.6084/m9.figshare.14265044) are publicly available on Figshare.
